# Does patient and public involvement influence the development of competency frameworks for the health professions? A systematic review

**DOI:** 10.3389/fmed.2022.918915

**Published:** 2022-07-26

**Authors:** Nicole Murray, Claire Palermo, Alan Batt, Kristie Bell

**Affiliations:** ^1^Monash Centre for Scholarship in Health Education, Faculty of Medicine, Nursing and Health Sciences, Monash University, Melbourne, VIC, Australia; ^2^Faculty of Medicine, Nursing and Health Sciences, Monash University, Melbourne, VIC, Australia; ^3^Department of Paramedicine, Faculty of Medicine, Nursing and Health Sciences, Monash University, Melbourne, VIC, Australia; ^4^Paramedic Programs, Faculty of Health Sciences and Human Services, Fanshawe College, London, ON, Canada; ^5^Department of Dietetics and Foodservices, Queensland Children's Hospital, Brisbane, QLD, Australia

**Keywords:** patient and public involvement, competency framework, health professions education, competency, competency development

## Abstract

Competency frameworks typically describe the perceived knowledge, skills, attitudes and other characteristics required for a health professional to practice safely and effectively. Patient and public involvement in the development of competency frameworks is uncommon despite delivery of person-centered care being a defining feature of a competent health professional. This systematic review aimed to determine how patients and the public are involved in the development of competency frameworks for health professions, and whether their involvement influenced the outcome of the competency frameworks. Studies were identified from six electronic databases (MEDLINE, CINAHL, PsycINFO, EMBASE, Web of Science and ERIC). The database search yielded a total of 8,222 citations, and 43 articles were included for data extraction. Most studies were from the United Kingdom (27%) and developed through multidisciplinary collaborations involving two or more professions (40%). There was a large variation in the number of patients and members of the public recruited (range 1–1,398); recruitment sources included patients and carers with the clinical condition of interest (30%) or established consumer representative groups (22%). Common stages for involving patients and the public were in generation of competency statements (57%) or reviewing the draft competency framework (57%). Only ten studies (27%) took a collaborative approach to the engagement of patients and public in competency framework development. The main ways in which involvement influenced the competency framework were validation of health professional-derived competency statements, provision of desirable behaviors and attitudes and generation of additional competency statements. Overall, there was a lack of reporting regarding the details and outcome of patient and public involvement. Further research is required to optimize approaches to patient and public involvement in competency framework development including guidance regarding who, how, when and for what purposes they should be engaged and the requirements for reporting.

**Systematic review registration:**

https://www.crd.york.ac.uk/prospero/, identifier: CRD42020203117.

## Introduction

Competency frameworks typically describe professional expectations of healthcare professionals by defining the perceived knowledge, skills, attitudes and other characteristics required to practice safely and effectively (1). They translate professional practice to activities such as assessment, curriculum design and educational frameworks, professional regulation, and clinical specialization (1–4). Despite this central role they play in developing and regulating professional practice (4), standardized approaches to developing competency frameworks are lacking (5). Uncertainty exists regarding which stakeholders to involve and for what purposes during the development of competency frameworks for health professions (2). A recent scoping review found significant variation in methodological approaches to competency framework development; a single, internationally recognized standard was not identified (5). A six-step model was recently proposed to improve the process, standard, reporting and evaluation of frameworks (1). While the model identified patients and members of the public as important stakeholders, it did not specify guidance on who, how, or for what purpose(s) to engage them.

Elsewhere in healthcare, there are a multitude of ways in which patients and the public are involved in shaping policy, services, and professional practice. Their involvement ranges from treatment decision making to health service development, evaluation, research and clinical practice guideline development (6). Much guidance exists regarding how, when and for what purpose(s) to engage patients and the public within these aspects of health care (7, 8). Involving patients and members of the public is advocated as it results in relevant and applicable recommendations that address patient preferences and needs, recognizes patients as experts in their health and illness, empowers patients in health care decisions, builds relationships with care providers and leads to development of person-centered and trustworthy services and guidelines (6). Within health professions education, there is a growing evidence base regarding patient and public involvement in teaching practices (9); however, their input into curriculum and competency framework development is less defined. While “patient-centered care” was reported as central in the development of most health professions frameworks in recent reviews, patient and public involvement during their development was lacking (2, 5). The specific methods used to engage patients and the public, the purpose of their engagement and the outcome of this engagement on the competency framework was beyond the scope of these reviews. Due to this lack of guidance, there remains uncertainty about how to best engage patients and the public in competency framework development processes and for what purpose(s).

Patients, caregivers, families, and members of the public are considered central stakeholders in the delivery of person-centered care (10); they bring different knowledge, needs, and concerns to a clinical encounter (11). Involving them as stakeholders in the development of competency frameworks enables their expectations of desirable knowledge, skills and attributes to be defined as observable behaviors and tasks that may be overlooked by health care professionals (11, 12). Authentically capturing this voice, alongside clinician input, could inform a competency framework that defines both patient and clinician expectations and supports training of the healthcare workforce to this standard (11). This has the potential to improve patient satisfaction with the care provided, establish positive relationships between healthcare services and their consumers and optimize patient outcomes and health as a result (13). Developing a competency framework without meaningful patient and public involvement may not adequately capture the complexities of person-centered care and may result in health professionals who are not competent to deliver care that truly meets the needs and preferences of this group (2, 11).

This systematic review aimed to determine how patients and the public are involved in the development of competency frameworks for health professions. More specifically, it aimed to answer the following research questions: What methods are used to involve patients and the public in the development of competency frameworks for health professions? Does patient and public involvement influence the outcome of competency framework development for health professions?

## Methods

### Definition and explanation of terms

In this study, “health professions” refer to those professions who “maintain health in humans through the application of the principles and procedures of evidence-based medicine and caring” (14). This includes implementation of preventive and curative measures, and promotion of health with the ultimate goal of meeting the health needs and expectations of individuals and groups (14).

“Competency” is defined as the observable ability of a health professional integrating knowledge, skills and attitudes in their performance of tasks in the workplace setting (3). A “competency framework” refers to the synthesis of these competencies into a structured framework that forms the requirements to practice in a particular clinical context.

In this study, “patients” refers to people (including children and adolescents) with lived experience of a health issue who access healthcare services or receive health care or advice. This includes the patient themselves and their family members or caregivers as well as the collective consumer groups that represent them (8, 15). “Public” refers to general members of the community including citizens and taxpayers; they may be potential users of health services but are not actively engaged in health care services (8, 15).

### Search strategy and eligibility

This study followed the format recommended in the Preferred Reporting Items for Systematic Reviews and Meta-Analysis (16). The study protocol was registered on 27 September 2020 on Prospero International Prospective Register of Systematic Reviews (study protocol: CRD42020203117).

In consultation with library staff, a search strategy was developed (see Supplementary Data). To develop the search strategy, we refined “health professions” to those who are registered or self-regulated to deliver health care. We used the professions listed with the Australian Health Practitioner Regulation Agency and National Alliance of Self-Regulating Health Professions as a reference point for developing search terms. Medical sub-specialties were identified through those approved by the Medical Board of Australia. Consultation with library staff indicated the professions identified in these reference materials were of international resonance. Search terms related to competency framework development were identified by scanning the key words and titles of studies included in a previous scoping review (5). Inclusion of key words related to patient and public involvement limited results to the exclusion of relevant studies and therefore were not included in the final search strategy. Six databases (MEDLINE, CINAHL, PsycINFO, EMBASE, Web of Science and ERIC) were identified that were most likely to yield relevant results. No limits were applied on publication date, study design or country of origin. Database searches were undertaken on 1 July 2020 and updated on 5 February 2022.

Studies were included if they:

(i) Reported methodology (quantitative, qualitative or mixed-methods approaches) used to develop competency frameworks for the health professions (undergraduate or postgraduate) pertaining to patient care; AND
(ii) Included one or more patients (adult, adolescent or child), family members or caregivers, collective consumer representative groups or members of the public; AND
(iii) Had undergone peer review and were published in English language.

Studies were excluded if they reported competency framework development for aspects of healthcare not involving individual patient care (e.g., health management, disaster management, public health).

### Study selection

Citations were managed in Endnote X9 (Clarivate Analytics, Philadelphia, PA) and duplicate papers were removed. The remaining abstracts were uploaded to Covidence (Veritas Health Innovation, Melbourne, Australia) for abstract screening. Each abstract and full text article was screened independently by two authors. NM screened all abstracts and full text articles; second author screening was undertaken by CP, AB and KB for approximately one third of the abstracts and full text articles each. Disagreements were resolved by discussion between at least two authors (NM and CP) with reference to the inclusion and exclusion criteria until consensus was achieved. A third author (AB or KB) was involved in discussions if consensus was unable to be reached.

### Data extraction and quality assessment

The Guidance for Reporting Involvement of Patients and the Public, Version 2 (GRIPP2) (17) was used as the quality assessment tool for the study as it is designed to assess the quality, consistency and reporting of patient and public involvement. The long form version (GRIPP2-LF) of the tool was selected for data extraction as it provided a comprehensive template. In addition to the elements listed in the GRIPP2-LF, article identification data (author details, year, and country of origin), health profession, focus of the competency framework, and whether the competencies were developed for entry-level or qualified health professionals were also extracted. The following additional fields were extracted as part of the GRIPP2-LF: (i) the inclusion of patient and public terms as key words in the abstract, (ii) the inclusion of a patient or member of the public as a co-author, and (iii) acknowledgment of patient and public participation. The data were compiled into a single spreadsheet in Microsoft Excel for extraction and synthesis.

Data were extracted from the included articles by NM. Where insufficient details regarding methodology were provided, attempts were made to contact the study authors. Thirty percent of the studies (*n* = 13) were selected for second author checking using an online random number generator. Approximately one third of these studies were allocated to CP, AB and KB for independent review of data extraction. Differences were resolved through discussions and the data extraction table updated accordingly.

### Data synthesis

Data were further categorized into methods used to involve patients and the public in the competency development process, total number of participants recruited, recruitment source (patients or caregivers with lived experience of the disease or condition, established consumer representative groups or members of the public) and whether demographics were reported (yes, no). The stage of involvement in the competency development process was also identified. The approach to patient and public involvement was classified using the criteria recommended by the National Institute for Health Research (7): consultation (user views inform health professional decision making), collaboration (forming an active partnership with users throughout the process) and user-controlled (controlled, directed and managed by service users). Narrative synthesis was used to summarize the outcome of patient and public involvement on the competency framework development. Key themes were identified and summarized as text.

The GRIPP2 short form (GRIPP2-SF) was considered the most appropriate for synthesizing and presenting data as it captured the key elements related to reporting of patient and public involvement in the included papers in a concise manner. Full marks were allocated for the methods (Section Methods) if the study included a clear description of the methods and stages that patient and public involvement was utilized, how participants were identified and recruited. Full marks were allocated for results (Section Discussion) if the study included the results of patient and public involvement and a description of how these results influenced the competency framework. Partial marks were allocated if they reported some of these criteria (but not all).

## Results

### Search results

**Figure 1:**
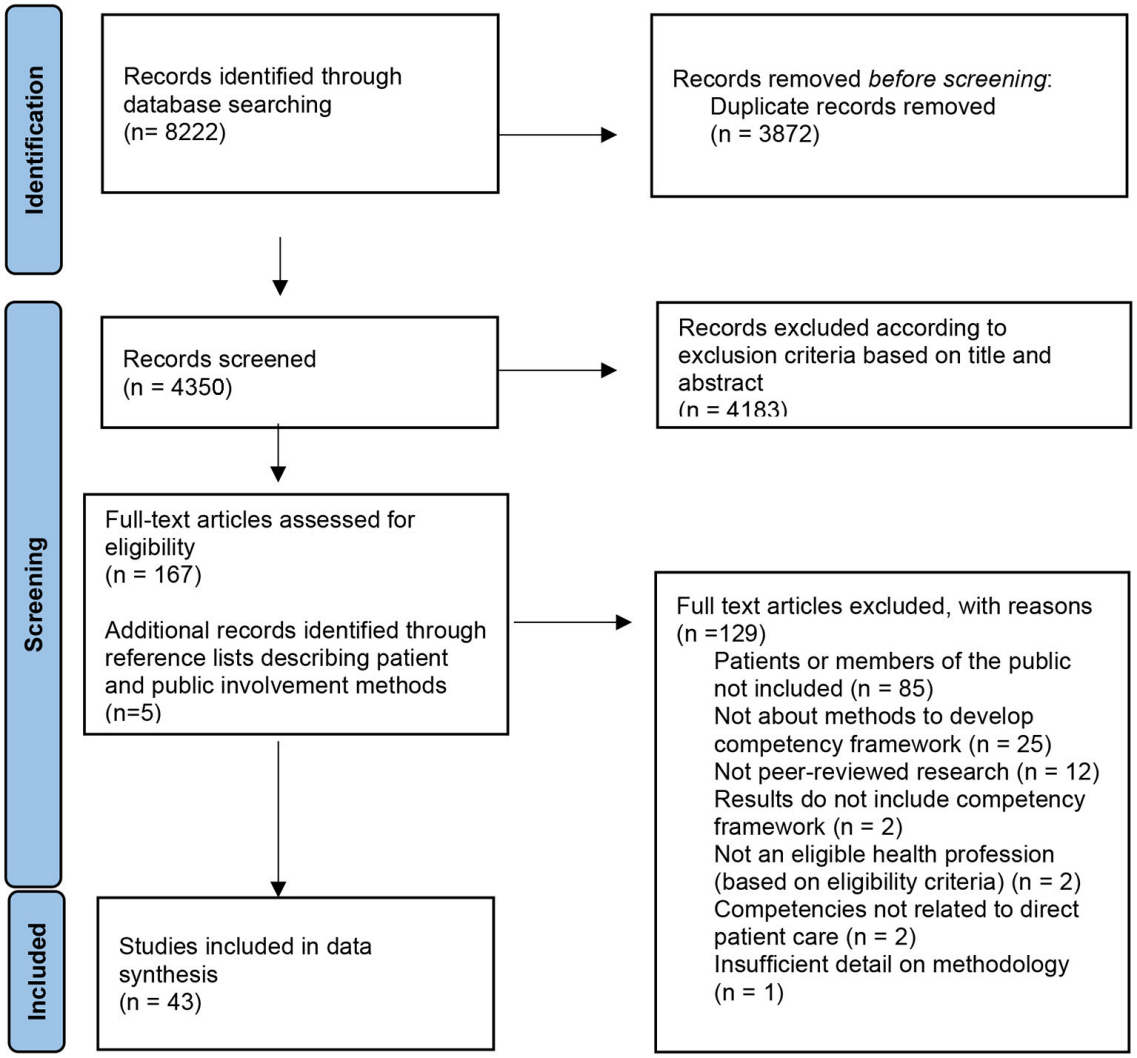
PRISMA flow diagram for identification of studies including patients and the public in competency framework development for health professions.

In total, the searches yielded 8,222 citations. Duplicate citations (*n* = 3,872) were removed, and a further 4,183 citations were excluded through title and abstract screening. After full text review of 167 citations, 37 papers met the inclusion criteria. Five additional papers describing detailed methods of patient and public involvement separately to the competency development paper were identified from the reference lists of these papers and included for data extraction. In total, 43 articles were included for data extraction, synthesis and quality assessment (Figure 1).

### Quality assessment

One paper (11) reported all five elements included on the GRIPP2-SF. Twelve (32%) studies did not adequately report any of the GRIPP-SF criteria. It was more common for studies to adequately report details regarding the methods (*n* = 17, 46%) than it was to discuss the results including the influence of patient and public involvement on the competency framework (*n* = 7, 19%) or to critically reflect on their involvement (*n* = 1, 3%). Fourteen (38%) studies partially reported details regarding the methods used for patient and public involvement; these studies provided details of the specific methods used (for example focus groups, interviews), however lacked detail regarding how the participants were identified and recruited. Eight (22%) studies partially reported involvement by summarizing the contribution of patients and the public but did not describe how they influenced the competency framework overall (Table 1).

**Table 1:** Quality assessment summary (columns 1–5: GRIPP2-SF; columns 6–8 added by authors).

**Author**	**Year**	**1. Aim**	**2. Methods**	**3.Study results**	**4. Discussion and conclusion**	**5. Reflection / critical perspective**	**6. Abstract / key words**	**7. Co-author (s)**	**8. Acknowledgments**
Anazodo et al. (18, 19)	2016 2019	✓^*^	✓	✓	✓				✓
Attard et al. (20)	2019		✓						✓
Barrett et al. (21) Rothen et al. (22)	2006 2007	✓^*^	✓	✓^*^	✓				✓^*^
Carter et al. (23)	2018		✓						
Dewing et al. (24)	2005								
Edelaar et al. (25)	2019								
El-Haddad et al. (11)	2017	✓	✓	✓	✓	✓	✓		
Erwin et al. (26, 27)	2018 (a) 2018 (b)	✓^*^	✓	^#^ ^*^			✓^*^		✓
Ferrier et al. (28)	2013		^#^						✓
Fields et al. (29)	2021		^#^	^#^	✓				✓
Gill et al. (30, 31)	2013 2014	✓^*^	✓^*^	^#^ ^*^			✓^*^		✓^*^
Hamburger et al. (32)	2015		^#^						
Haruta et al. (33)	2016		^#^						
Hill et al. (34, 35)	2011 (a) 2011 (b)								
Hinman et al. (36)	2020	✓	✓					✓	
Homer et al. (37)	2012								
Homer et al. (38, 39)	2007 2009	✓^*^	✓^*^	^#^ ^*^					✓
Huth et al. (40)	2021		^#^					✓	
Kirk et al. (41)	2013		✓						
McCallum et al. (42)	2018								
Mills et al. (4)	2021	✓	✓	✓					
Parmar et al. (43)	2021		^#^				✓		
Roche et al. (44)	2019		✓	✓					
Scott et al. (45)	2003		^#^						
Skirton et al. (46)	2010		^#^						
Smith et al. (47)	2011		✓	✓					
Smythe et al. (48)	2014		^#^	^#^					
Stanyon et al. (49)	2017		^#^						✓
Van der Aa et al. (10)	2020		✓	✓					
Walker et al. (50)	2016		✓	^#^					
Walpole et al. (51)	2016		^#^						
Warnock et al. (52)	2013		^#^	^#^					
Witt et al. (53)	2022								
Xing et al. (54)	2019		^#^						
Yang et al. (55)	2013		✓						
Yates et al. (56)	2007		^#^						
Young et al. (57)	2000		✓	^#^					✓

* *Detail included in consumer methodology paper, but not the competency development methodology paper*.

# *Partially reported*.

*The ✓indicates sufficient details provided*.

### Study characteristics

Most studies were from the United Kingdom (*n* = 10, 27%), with others from across Europe (*n* = 7, 19%), Australia (*n* = 8, 22%), Canada (*n* = 3, 8%), the United States of America (*n* = 3, 8%), China (*n* = 2, 5%) and Japan (*n* = 1, 3%). Three studies (8%) were international collaborations involving data collection from two or more countries. Ten (27%) of the studies were from the profession of nursing, six (16%) were from medicine, two (5%) from midwifery and there were one (3%) each from psychology, occupational therapy, and genetic counseling. One study (3%) was for the professions of nursing and midwifery. Fifteen (40%) were multidisciplinary collaborations including two or more health professions. Three (8%) of studies developed competencies for entry level health professionals; the remainder (*n* = 34, 92%) were developed for qualified health professionals (Table 2).

**Table 2:** Study characteristics and methods used for patient and public involvement.

**Lead author**	**Year**	**Location**	**Profession**	**Competency** **focus**	**Practice level**	**Focus** **groups**	**Interviews**	**Survey**	**Consensus** **methods** **(Delphi** **Process)**	**Consensus** **methods** **(Other)**	**Nominal** **group** **technique**	**Workshop/** **symposium**	**Insufficient** **detail**
Anazodo et al. (18, 19)	2016 2019	Australia	Multidisciplinary	Oncofertility	Qualified	X			X				
Attard et al. (20)	2019	Europe	Nursing and midwifery	Spiritual care	Entry level	X			X				
Barrett et al. (21) Rothen et al. (22)	2006 2007	Europe	Medicine	Intensive care	Qualified		X	X					
Carter et al. (23)	2018	United Kingdom	Nursing	Admiral nursing	Qualified	X							
Dewing et al. (24)	2005	United Kingdom	Nursing	Admiral nursing	Qualified		X						
Edelaar et al. (25)	2019	Europe	Multidisciplinary	Rheumatology	Qualified								X
El-Haddad et al. (11)	2017	Australia	Medicine	Acute lower back pain	Qualified		X	X					
Erwin et al. (27) Erwin et al. (26)	2018 2018	United Kingdom	Multidisciplinary	Arthritis	Qualified	X		X	X				
Ferrier et al. (28)	2013	Canada	Genetic counseling	Genetic counseling	Qualified			X					
Fields et al. (29)	2021	Australia	Occupational therapy	Driver assessors	Qualified	X							
Gill et al. (30, 31)	2013 2014	Australia	Nursing	Intensive care	Qualified	X	X						
Hamburger et al. (32)	2015	United States	Medicine	Pediatrics	Qualified	X							
Haruta et al. (33)	2016	Japan	Multidisciplinary	Interprofessional practice	Entry level			X				X	
Hill et al. (34, 35)	2011 (a) 2011 (b)	Europe	Nursing	Diabetes	Qualified								X
Hinman et al. (36)	2020	International	Multidisciplinary	Osteoarthritis	Qualified				X				
Homer et al. (38, 39)	2007 2009	Australia	Midwifery	Midwifery care	Qualified			X					
Homer et al. (37)	2012	Australia	Midwifery	Midwifery care	Qualified			X					
Huth et al. (40)	2021	United States	Medicine	Pediatrics	Qualified	^#^				X			
Kirk et al. (41)	2013	United Kingdom	Nursing	Genetics	Qualified			X			X		
McCallum et al. (42)	2018	Canada	Multidisciplinary	Palliative care	Qualified								X
Mills et al. (4)	2021	International	Multidisciplinary	Rehabilitation	Qualified			X					
Parmar et al. (43)	2021	Canada	Multidisciplinary	Family Care Giver	Qualified				X			X	
Roche et al. (44)	2019	Australia	Psychology	Vision impairment	Qualified		X						
Scott et al. (45)	2003	Europe	Multidisciplinary	Diabetes	Qualified			X					
Skirton et al. (46)	2010	Europe	Multidisciplinary	Genetics	Qualified			X					
Smith et al. (47)	2011	United Kingdom	Multidisciplinary	Pediatrics	Qualified	X							
Smythe et al. (48)	2014	United Kingdom	Multidisciplinary	Dementia care	Qualified	X		X					
Stanyon et al. (49)	2017	United Kingdom	Nursing	Aged care	Qualified	X							
Van der Aa et al. (10)	2020	Europe	Medicine	Obstetrics and Gynecology	Qualified			X		X			
Walker et al. (50)	2016	United Kingdom	Multidisciplinary	Breech births	Qualified				X				
Walpole et al. (51)	2016	United Kingdom	Medicine	Global health	Qualified		X	X					
Warnock et al. (52)	2013	United Kingdom	Nursing	Oncology (late effects)	Qualified								X
Witt et al. (53)	2022	International	Multidisciplinary	Oncology (integrative)	Qualified				X	X			
Xing et al. (54)	2019	China	Nursing	Diabetes	Qualified							X	
Yang et al. (53)	2013	China	Nursing	General nursing	Entry level			X					
Yates et al. (56)	2007	Australia	Nursing	Oncology (Breast care)	Qualified			X					
Young et al. (57)	2000	United States	Multidisciplinary	Mental health	Qualified	X	X	X		X			

# *Facilitated focus groups as a member of the study team*.

*The X symbol indicates the study used that method or involved consumers at that stage*.

### Recruitment methods

There was a large variation in the numbers of patients and public members recruited, ranging from 1 to 1,398. Twelve (32%) studies did not report how many participants were included. Eight (21%) studies reported the demographics of the participants included in the competency development process. The most common sources of recruitment were patients and/or carers with the clinical condition of interest (*n* = 12, 32%) or established consumer representative groups (*n* = 8, 22%). Five studies (14%) used a combination of these two sources. One study (3%) recruited members of the public (33). Eleven (30%) studies did not report how or where they recruited their participants from Table 3.

**Table 3:** Summary of patient and public involvement in development of competencies.

**Lead Author**	**Total combined number involved**	**Patient demographics reported**	**Recruitment source**	**Approach to patient and public involvement**	**Generation of competency statements**	**Review draft competency statement**	**Consensus to finalize competency statements**	**Reference group involvement**	**Co-author manuscript**	**Not reported**
Anazodo et al. (18, 19)	*n* = 157	Yes	Consumer group	Collaborative	X		X			
Attard et al. (20)	*n* = 57	No	Patients/carers Consumer group	Consultative	X		X			
Barrett et al. (21) Rothen et al. (22)	*n* = 1,398	Yes	Patients/carers	Consultative	X					
Carter et al. (23)	*n* = 6	No	Consumer group	Consultative	X	X		X		
Dewing et al. (24)	NR	No	Patients/carers	Consultative	X					
Edelaar et al. (25)	*n* = 3	No	NR	Consultative				X		
El-Haddad et al. (11)	n = 14	Yes	Patients/carers	Collaborative	X	X				
Erwin et al. (26, 27)	*n* = 28	Yes	Patients/carers	Collaborative	X		X			
Ferrier et al. (28)	*n* = 3	No	Patients/carers Consumer group	Consultative		X				
Fields et al. (29)	*n* = 13	No	Consumer group	Consultative		X				
Gill et al. (30, 31)	*n* = 17	Yes	Consumer group	Consultative	X					
Hamburger et al. (32)	NR	No	NR	Consultative	X					
Haruta et al. (33)	NR	No	General public	Consultative		X				
Hill et al. (34, 35)	NR	No	NR	Consultative	X	X				
Hinman et al. (36)	*n* = 27	No	Patients/carers Consumer group	Collaborative		X	X	X	X	
Homer et al. (37)	NR	No	NR	Consultative		X		X		
Homer et al. (38, 39)	*n* = 28	Yes	Patients/carers Consumer group	Consultative						X
Huth et al. (40)	*n* = 2	No	Patients/carers	Collaborative	X	X		X	X	
Kirk et al. (41)	*n* = 4	No	Patients/carers	Collaborative	X	X				
McCallum et al. (42)	*n* = 1	No	NR	Consultative						X
Mills et al. (4)	*n* = 36	Yes	Patient/carers Consumer group	Consultative		X				
Parmar et al. (43)	*n* = 8	No	NR	Collaborative	X	X	X			
Roche et al. (44)	*n* = 5	Yes	Patients/carers	Consultative	X					
Scott et al. (45)	NR	No	NR	Consultative		X				
Skirton et al. (46)	NR	No	Consumer group	Consultative		X				
Smith et al. (47)	*n* = 47	No	Patients/carers	Consultative	X					
Smythe et al. (48)	NR	No	NR	Consultative	X					
Stanyon et al. (49)	*n* = 3	No	Patients/carers	Consultative	X					
Van der Aa et al. (10)	NR	No	Consumer group	Collaborative	X	X		X		
Walker et al. (50)	*n* = 2	No	Consumer group	Collaborative	X		X			
Walpole et al. (51)	NR	No	NR	Consultative		X	X			
Warnock et al. (52)	NR	No	NR	Consultative		X				
Witt et al. (53)	NR	No	NR	Consultative		X	X			
Xing et al. (54)	*n* = 5	No	Patients/carers	Consultative		X				
Yang et al. (55)	*n* = 185	No	Patients/carers	Consultative			X			
Yates et al. (56)	*n* = 1	No	Consumer group	Consultative	X	X				
Young et al. (57)	*n* = 45	No	Patients/carers	Collaborative	X	X	X			

*NR, Not reported*.

*The X symbol indicates the study used that method or involved consumers at that stage*.

### Engagement methods and approach

Almost half of the studies (*n* = 15, 40%) used more than one method in the competency framework development process. The most common method used was survey (*n* = 17, 46%). Other methods included focus groups (*n* = 11, 30%), interviews (*n* = 7, 19%), Delphi processes (*n* = 4, 11%), other consensus methods (*n* = 4, 11%), nominal group techniques (*n* = 1, 2%) or workshops and symposiums (*n* = 3, 8%). Three studies (8%) did not provide enough detail to determine the methods used (Table 2).

Ten (27%) studies utilized a collaborative approach to competency framework development, whereby the participants could be described as having an active partnership throughout the process (6). Consultative approaches were more common (*n* = 27, 73%), whereby the opinions of patients or members of the public were sought but health professionals remained the overall decision makers. There were no user-controlled approaches to the competency framework development process. Three studies engaged patients indirectly. Dewing and Traynor (24) and Homer et al. (38) included observation of clinical practice involving patient care, where the focus of observation was the health professional's competence rather than the patient's perspective of competence. Kirk et al. (41) utilized real patient stories as an anchor for the nominal group technique discussions involving both health professionals and patients. Stakeholder groups received the stories alongside the competency framework and were asked to firstly consider the patient needs and secondly what the nurse needed to know, think, and do to meet these needs (41).

### Stage of competency development process

Seventeen (46%) of the studies involved patients or the public in more than one stage of the competency development process. Most studies involved patients or the public in the generation of competency statements (*n* = 21, 57%) or in review of draft competency statements (*n* = 21, 57%). Patients or members of the public were also included in consensus methods to finalize competency statements (*n* = 10, 27%), involved in the project reference group or steering committee (*n* = 6, 16%) and co-authoring the manuscript (*n* = 2, 5%). Two studies (5%) did not include enough detail to determine which stage of the process the patients or members of the public were involved in Table 3.

### Outcome of engagement

Fifteen (40%) studies provided a summary of the results of patient and public engagement in the competency development process; four of the studies reported these results in a separate paper (Table 3). Patients and members of the public indicated that health professionals need to have current and evidence-based knowledge, skill and expertise in the assessment and treatment of the clinical condition (4, 21, 22, 26, 39, 47). Effective communication skills were also emphasized (4, 26, 30, 39, 47, 48); this included the ability to explain diagnosis and treatment plans (11) and the impact of treatment on their future health (18). Patients wanted to be an active participant in their care (11, 21, 22, 26, 39) and for their unique personal circumstances to be considered in care planning (11, 26, 30, 47). The ability of health professionals to provide psychosocial support was highlighted (18, 30). Patients highlighted the importance of involving, coordinating, or referring to other relevant health professionals and services (11, 26, 32, 39, 47), including assisting with navigating the health care system (32) and patient advocacy (30). They indicated a holistic approach to care was preferred (26, 47), involving families and caregivers where relevant (4, 47). Other desirable qualities such as being respectful (11, 48), compassionate (11), empathetic (48), approachable (47), kind (48) and creating a warm and safe environment (30, 48) were described.

### Outcome of involvement on competency framework

Of the 14 studies that described the outcome of patient and public involvement, seven provided a further description of how these results were incorporated into the competency framework. There were three main ways that patient and public involvement influenced the competency framework: validation or triangulation of competency statements, defining desirable behaviors and attributes (non-technical skills), and generation of additional competency statements.

Several studies described the way in which patients or members of the public validated or triangulated the competencies proposed by health professionals. For example, Anazodo et al. (18) involved consumers in a consensus process to determine the final competency statements. They reported broad agreement between health care professionals and patients for most competency statements. Mills et al. (4) also reported agreement between health professionals and patients with the proposed core values, core beliefs, practice and professionalism competencies. Rothen et al. (22) surveyed a large cohort of 1,398 patient and relatives post discharge from European intensive care units. The survey contained 21 statements outlining characteristics of medical competence; participants ranked them all as either important or essential in a similar fashion to health professionals who were surveyed. Roche and Chur-Hansen (44) incorporated the views of participants with vision impairment to triangulate the competencies proposed by health professionals; all themes from which the competencies were derived incorporated the perspective of both groups. El-Haddad et al. (11) clearly identified where the views of patients and health professionals were similar and unique in the development of entrustable professional activities (EPA) for lower back pain management. Patient input was incorporated into each component of the EPA descriptors except for “knowledge” and “skills”.

Patients and members of the public also provided desirable and observable descriptions of health professional behaviors and attitudes which inform the non-technical skill component of the frameworks. This included contribution to the development of competencies regarding communication and professionalism. Smith et al. (47) described attributes that were defined by children, young people and parents; these attributes were reported as integral to the behavioral components and included in their definition of competence and competence descriptors. Van der Aa et al. (10) engaged patient representative groups who described four elements important to their care: basic connection skills, individualized care, informed choice, and attention to setting and context. These elements were integrated into two (of four) competency domains (“Patient-Centered Care” and “Systems Based Practice”). El-Haddad et al. (11) included specific desirable and observable behaviors and attitudes that were primarily informed by patient involvement. These attitudes are clearly articulated in the final EPA statement and include, for example, empathetic and understanding communication style and compassionate care. The EPAs also integrated attitudes identified as important by patients including seeking appropriate supervision and addressing patient concerns and priorities. Rothen et al. (22) incorporated qualitative comments from their consumer survey into the “Professionalism” domain of their competency framework including communication, professional relationships with patients and relatives, and self-governance as a health care professional.

Finally, patient and public involvement also resulted in generating additional competency statements, or re-phrasing statements proposed by health professionals. In the study by Mills et al. (3), consumers felt that the collaborative nature of rehabilitation care was not clearly articulated and represented; therefore, an additional core belief was added to framework to rectify this. Anazodo et al. (18) found a disagreement in expectations between health care professionals and consumers regarding the timing of referral to fertility services. Once the statement was re-phrased to remove specific timeframes, the two groups reached agreement and the competency was included in the final framework. El-Haddad et al. (11) clearly identified the contribution of patients in shaping the final EPAs for management of patients with lower back pain; this included communicating diagnosis and pathology, discussing the role of spinal imaging, and informing the patient of the interprofessional team's management plan.

## Discussion

This study aimed to determine how patients and the public are involved in competency framework development and how their involvement influenced the outcome of the framework. It builds on previous work that identified a lack of patient and public involvement in competency framework development, despite most frameworks stating a “patient-centered” focus (2, 5). This study found variations in the recruitment, approaches, and methods used to engage patients, and through its synthesis provides recommendations for methods to ensure patient and public voices are truly heard, represented, and adequately reported.

Overall, the justification for patient and public involvement and how participants were identified and recruited was poorly reported. There was large variation in the number of patients recruited; most studies did not report the characteristics of participants. Due to this lack of detail, it was difficult to determine if the participants represented the many diverse perspectives of users of health professional care. For example, the needs and preferences of all women receiving midwife care was not adequately represented as the majority voice was women who had home birthed (39). Considerations of cultural diversity, gender, age, social circumstances, race, sexuality, health literacy, communication challenges and groups that are difficult to reach were not discussed or actively targeted in any of the studies included. Such groups may have different or greater healthcare needs and require different approaches compared with the wider population (13). Excluding their needs and preferences from the consultation process represents a missed opportunity to develop health professionals that can competently deliver their care. Several studies included only a select few individuals in the development process [for example: (25, 28, 40, 42, 49, 50, 56)]; this is unlikely to highlight the diverse needs and preferences for care or create meaningful input. Even for the studies that included a larger group of patients, they were often small in comparison to the number of health professionals involved in the competency framework development. Majid (58) describes “unequal power” and “limited impact” as two dimensions of tokenism in patient and public engagement; arguably the limited number of participants involved in some studies represents such dimensions of tokenism. Involving only a few individuals, or a proportionally small number, of patients or public not only risks limiting representativeness, but also decreases the strength of their opinion which may be particularly important when their perspectives differ to the health professionals involved (6). Finally, we only identified one study that included members of the public. Patients and members of the public have distinct and different roles in health care decision making and value health states differently (15). Patients draw on lived experience as a health service user and contribute their individual perspective, whereas members of the public draw on collective aspirations and the broader public interest (15). It is reasonable to assume they would bring different perspectives and influence on the competency development process. Together, these findings indicate there is a greater need to consider diversity and inclusivity in involvement of patients and members of the public when developing competency frameworks to ensure their voices are truly heard, represented, and adequately justified and reported. Improved guidance regarding how to target populations that are hard to reach and typically under-represented may help strengthen this process.

The most common stages for patient and public involvement were in the generation of competency statements, and to provide feedback on the draft competency framework that health professional groups had developed. Common methods to achieve this included focus groups, interviews, and surveys; these are considered active approaches that improve patient and public involvement when compared to more passive approaches such as public comment (6). Through these approaches, the health professionals retained the power to make the decision as to whether the competencies generated through patient and public involvement were included in the final framework or not. Such approaches are considered consultative due to the unidirectional flow of information from patients and the public to the health professionals (6). Consultative approaches are positioned on the lower end of the engagement continuum as patients and the public are involved but have limited power or decision-making ability (13). This power imbalance also lends itself to criticism of tokenism, where patient and public engagement is utilized to maintain existing decisions rather than generating new ideas (58). Improved guidance on how and when to engage patient and members of the public in the competency development process may help shift this power balance to enable the patient voice to be incorporated in a meaningful and genuine way.

By contrast, collaborative approaches to patient and public involvement involve a bidirectional information exchange where they are considered active participants in the decision-making processes (6). This develops a collective perspective incorporating the patient voice (6) and empowers patients at the individual level (13). Involving patients collaboratively also contributes to a culture of person-centredness throughout the development process, allowing patient-relevant outcomes to be identified (12). El-Haddad et al. (11) demonstrated how patient involvement can both identify areas overlooked by clinicians and also complement their perspective. Other collaborative approaches included involvement in multiple stages of the development process such as competency statement generation and consensus techniques [example: (10, 27, 36, 40, 41, 43, 50, 57)] or inclusion in project references groups overseeing the progress of the whole development process [example: (10, 23, 25, 36, 37)]. There was a lack of critical evaluation of these collaborative approaches making it difficult to determine the most effective way to use these methods in the future. It is perhaps unsurprising that there were no user-controlled approaches to competency framework development in this review given that a necessary component is the description of professional practice, knowledge, and skills. However, there is scope to move toward co-created approaches that give the patients more power in decision-making processes and to enable their needs and preferences to be heard as an equal voice.

We identified a lack of clear reporting of patient and public involvement, particularly regarding the results, outcome and evaluation of involvement including the influence on the competency framework overall. In contrast to this inadequate reporting of patient and public involvement, most studies described the involvement of health professionals in detail, including recruitment sources, methods used to engage them in the process and the outcome of their involvement. Several studies combined the results of health professional and patient involvement, and therefore the contribution of patients and members of the public to the process could not be delineated. The lack of reporting and evaluation of patient and public involvement in the competency development makes it difficult to determine who, how, when, and for what purpose(s) patients and the public should be involved in the competency framework development process. Reporting guidelines clearly articulate the need to report sampling details regardless of methods and this should apply for patients and members of the public involved in research. This lack of detail may reflect the lack of clear guidance for health professionals on how to involve patients and the public when developing competency frameworks.

### Strengths and limitations

This review provides a comprehensive synthesis of patient and public involvement in competency framework development processes; the conclusions should be considered in view of the methodological limitations. The review utilized broad search criteria that were not limited to patient and public involvement search terms to identify all relevant literature. Despite this, it is possible relevant studies were missed, particularly if the inclusion of patient and public involvement was not identified in the title or abstract and excluded at this stage of screening. It is also possible relevant literature was excluded if it was not published in the English language or had not undergone peer-review. Internationally, health care systems, health professions and competency standard terminology are heterogenous. Despite the use of broad search criteria, we may have excluded relevant literature because of the terms selected. Finally, the articles included in this review encompass broad perspectives across the health care spectrum. Involvement of patients in delivery of care may vary across these practice areas, and therefore the expectations of their involvement as a key stakeholder group in competency framework development may also vary.

## Conclusion

Patients and members of the public bring different needs, preferences, and perspectives to a clinical encounter. To define a truly person-centered approach and equip the future healthcare workforce to provide this care, their involvement in the competency framework development process is desirable. Further research is needed to identify optimal approaches for patient and public involvement and how best to align clinician, patient and public needs and expectations in the context of developing competency frameworks. Collaborative and co-design approaches, which allow the patient voice to be heard equally in the decision-making process, should be further explored and evaluated in order to understand how these influence competency frameworks. Guidance on who, how, when and for what purpose(s) patients and the public should be engaged in the competency framework development process is required, including clear reporting of outcomes and critical reflections to guide future approaches. Future research could also evaluate the impact of competency frameworks developed using collaborative approaches on person-centered practice and health care delivery.

## Data availability statement

The original contributions presented in the study are included in the article/Supplementary material, further inquiries can be directed to the corresponding author.

## Author contributions

NM, CP, AB, and KB contributed to the study concept and design, abstract and full text screening, and review of data extraction. NM developed and conducted literature searches and performed data extraction. All authors revised and approved the final manuscript.

## Conflict of interest

The authors declare that the research was conducted in the absence of any commercial or financial relationships that could be construed as a potential conflict of interest.

## Publisher's note

All claims expressed in this article are solely those of the authors and do not necessarily represent those of their affiliated organizations, or those of the publisher, the editors and the reviewers. Any product that may be evaluated in this article, or claim that may be made by its manufacturer, is not guaranteed or endorsed by the publisher.
