# Supplementary material for: Does patient and public involvement influence the development of competency frameworks for the health professions? A systematic review
Source: Front Med (Lausanne). 2022 Jul 26;9:918915. doi: 10.3389/fmed.2022.918915 (PMC9360578; doi:10.3389/fmed.2022.918915)
Supplement: Supplementary file 1 [file Data_Sheet_1.docx]

**Supplementary data: Search Strategy**

**Medline, Psychinfo, and Embase via Ovid**

(medicine or medical Or physician* Or pediatric* or paediatric* Or anaesthet* or dermatolog* Or general practi* Or intensivist* or critical care or oncolog* Or obstetric* Or gynaecolog* or gynecolog* Or opthamolog* Or neonatolog* Or cardiolog* Or endocrinolog* Or genetic* or gastroenterolog* Or haematolog* or hematolog* Or immunolog* Or allerg* Or nephrolog* Or neurolog* or rheumatolog* Or patholog* Or hepatolog* Or geriatric* Or psychiatr* Or radiolog* Or nuclear medicine or surg* Or neurosurg* Or urolog* Or Nurs* or midwif* Or midwives Or art therap* or audiolog* or radiograph* or radiation therap* or radiotherap* or imaging technolog* Or ultrasonograph* or sonograph* or dieti?ian* or dietetic* or exercise physiolog* or music therap* or occupational therap* or optometr* or orthopt* or orthoti* or prostheti* or osteopath* or perfusionist* or physiotherap* or physical therap* or podiatr* or psycholog* or rehabilitation counsel* or social work* or speech therap* or speech patholog* or dent* or paramed* Or emergency medical technician* or pharmac*).ti,ab.

**OR**

exp Physicians/ or Anesthetists/ or Emergency Medicine/ or Genetics, Medical/ or Hematology/ or Neurology/ or Palliative Medicine/ or exp Psychiatry/ or Radiology/ or exp Specialties, Surgical or exp Nurses/ or exp Specialties, Nursing/ or Art Therapy/ or Audiologists/ or Radiotherapy/ or Diagnostic Imaging/ or Ultrasonography/ or Nutritionists/ or Genetic Therapy/ or Music Therapy/ or Occupational Therapists/ or Optometrists/ or Osteopathic Physicians/ or Physical Therapists/ or Podiatry/ or exp Psychology/ or Social Workers/ or Speech-Language Pathology/ or Dentists/ or Emergency Medical Technicians/ or Pharmacists/

**AND**

((develop* or produc* or map* or construct* or consensus or establish* or determin* or validat* or identif* or build* or implement* or defin* or introduc*) adj4 (entrustable professional activit* or (framework* adj3 competenc*) or (standard* adj3 competenc*) or (profile adj3 competenc*) or (standard* adj3 practice*) or core competenc* or competency model* or capability framework*)).ti,ab.

**Cinahl via Ebscohost**

AB ((develop* or produc* or map* or construct* or consensus or establish* or determin* or validat* or identif* or build* or implement* or defin* or introduc*) N4 (“entrustable professional activit*” or (framework* N3 competenc*) or (standard* N3 competenc*) or (profile* N3 competenc*) or “core competenc*” or “competency model*” or “capability framework*”))

OR

TI ((develop* or produc* or map* or construct* or consensus or establish* or determin* or validat* or identif* or build* or implement* or defin* or introduc*) N4 (“entrustable professional activit*” or (framework* N3 competenc*) or (standard* N3 competenc*) or (profile* N3 competenc*) or “core competenc*” or “competency model*” or “capability framework*”))

AND

AB (medicine or medical Or physician* Or pediatric* or paediatric* Or anaesthet* or dermatolog* Or “general practi*” Or intensivist* or “critical care” or oncolog* Or obstetric* Or gynaecolog* or gynecolog* Or opthamolog* Or neonatolog* Or cardiolog* Or endocrinolog* Or genetic* or gastroenterolog* Or haematolog* or hematolog* Or immunolog* Or allerg* Or nephrolog* Or neurolog* or rheumatolog* Or patholog* Or hepatolog* Or geriatric* Or psychiatr* Or radiolog* Or “nuclear medicine” or surg* Or neurosurg* Or urolog* Or Nurs* or midwif* Or midwives Or “art therap*” or audiolog* or radiograph* or “radiation therap*” or radiotherap* or “imaging technolog*” Or ultrasonograph* or sonograph* or dieti?ian* or dietetic* or “exercise physiolog*” or “music therap*” or “occupational therap*” or optometr* or orthopt* or orthoti* or prostheti* or osteopath* or perfusionist* or physiotherap* or “physical therap*” or podiatr* or psycholog* or “rehabilitation counsel*” or “social worker*” or “speech therap*” or “speech patholog*” or dent* or paramed* Or “emergency medical technician*” or pharmac*)

OR

TI (medicine or medical Or physician* Or pediatric* or paediatric* Or anaesthet* or dermatolog* Or “general practi*” Or intensivist* or “critical care” or oncolog* Or obstetric* Or gynaecolog* or gynecolog* Or opthamolog* Or neonatolog* Or cardiolog* Or endocrinolog* Or genetic* or gastroenterolog* Or haematolog* or hematolog* Or immunolog* Or allerg* Or nephrolog* Or neurolog* or rheumatolog* Or patholog* Or hepatolog* Or geriatric* Or psychiatr* Or radiolog* Or “nuclear medicine” or surg* Or neurosurg* Or urolog* Or Nurs* or midwif* Or midwives Or “art therap*” or audiolog* or radiograph* or “radiation therap*” or radiotherap* or “imaging technolog*” Or ultrasonograph* or sonograph* or dieti?ian* or dietetic* or “exercise physiolog*” or “music therap*” or “occupational therap*” or optometr* or orthopt* or orthoti* or prostheti* or osteopath* or perfusionist* or physiotherap* or “physical therap*” or podiatr* or psycholog* or “rehabilitation counsel*” or “social worker*” or “speech therap*” or “speech patholog*” or dent* or paramed* Or “emergency medical technician*” or pharmac*)

OR

MH (exp Physicians/ or Anesthetists/ or Emergency Medicine/ or Genetics, Medical/ or Hematology/ or Neurology/ or exp Psychiatry/ or exp Specialties, Surgical or exp Nurses/ or exp Specialties, Nursing/ or Art Therapy/ or Audiologists/ or Radiotherapy/ or Diagnostic Imaging/ or Ultrasonography/ or Dietitians/ or Radiology Personnel/ or Music Therapy/ or Occupational Therapists/ or Optometrists/ or Osteopaths/ or Physical Therapists/ or Podiatry/ or Psychology/ or Social Workers/ or Speech-Language Pathology/ or Dentists/ or Emergency Medical Technicians/ or Pharmacists/)

**ERIC via Proquest**

AB (develop* or produc* or map* or construct* or consensus or establish* or determin* or validat* or identif* or build* or implement* or defin* or introduc*) NEAR/4 (“entrustable professional activit*” or (framework* NEAR/3 competenc*) or (standard* NEAR/3 competenc*) or (profile* NEAR/3 competenc*) or “core competenc*” or “competency model*” or “capability framework*”)

OR

TI (develop* or produc* or map* or construct* or consensus or establish* or determin* or validat* or identif* or build* or implement* or defin* or introduc*) NEAR/4 (“entrustable professional activit*” or (framework* NEAR/3 competenc*) or (standard* NEAR/3 competenc*) or (profile* NEAR/3 competenc*) or “core competenc*” or “competency model*” or “capability framework*”)

AND

AB (medicine or medical Or physician* Or pediatric* or paediatric* Or anaesthet* or dermatolog* Or “general practi*” Or intensivist* or “critical care” or oncolog* Or obstetric* Or gynaecolog* or gynecolog* Or opthamolog* Or neonatolog* Or cardiolog* Or endocrinolog* Or genetic* or gastroenterolog* Or haematolog* or hematolog* Or immunolog* Or allerg* Or nephrolog* Or neurolog* or rheumatolog* Or patholog* Or hepatolog* Or geriatric* Or psychiatr* Or radiolog* Or “nuclear medicine” or surg* Or neurosurg* Or urolog* Or Nurs* or midwif* Or midwives Or “art therap*” or audiolog* or radiograph* or “radiation therap*” or radiotherap* or “imaging technolog*” Or ultrasonograph* or sonograph* or dieti?ian* or dietetic* or “exercise physiolog*” or “music therap*” or “occupational therap*” or optometr* or orthopt* or orthoti* or prostheti* or osteopath* or perfusionist* or physiotherap* or “physical therap*” or podiatr* or psycholog* or “rehabilitation counsel*” or “social worker*” or “speech therap*” or “speech patholog*” or dent* or paramed* Or “emergency medical technician*” or pharmac*)

OR

TI (medicine or medical Or physician* Or pediatric* or paediatric* Or anaesthet* or dermatolog* Or “general practi*” Or intensivist* or “critical care” or oncolog* Or obstetric* Or gynaecolog* or gynecolog* Or opthamolog* Or neonatolog* Or cardiolog* Or endocrinolog* Or genetic* or gastroenterolog* Or haematolog* or hematolog* Or immunolog* Or allerg* Or nephrolog* Or neurolog* or rheumatolog* Or patholog* Or hepatolog* Or geriatric* Or psychiatr* Or radiolog* Or “nuclear medicine” or surg* Or neurosurg* Or urolog* Or Nurs* or midwif* Or midwives Or “art therap*” or audiolog* or radiograph* or “radiation therap*” or radiotherap* or “imaging technolog*” Or ultrasonograph* or sonograph* or dieti?ian* or dietetic* or “exercise physiolog*” or “music therap*” or “occupational therap*” or optometr* or orthopt* or orthoti* or prostheti* or osteopath* or perfusionist* or physiotherap* or “physical therap*” or podiatr* or psycholog* or “rehabilitation counsel*” or “social worker*” or “speech therap*” or “speech patholog*” or dent* or paramed* Or “emergency medical technician*” or pharmac*)

OR

MAINSUBJECT (exp Physicians/ or Emergency Medicine/ or Genetics, Medical/ or Neurology/ or Physical and Rehabilitation Medicine/ or exp Psychiatry/ or Radiology/ or Nurses/ or Art Therapy/ or Dietitians/ or Genetic Therapy/ or Music Therapy/ or Occupational Therapists/ or Optometrists/ or Physical Therapists/ or Podiatry/ or exp Psychology/ or Social Workers/ or Speech-Language Pathology/ or Dentists/ or Emergency Medical Technicians/ or Pharmacists/)

**Web of Science**

AB= ((develop* or produc* or map* or construct* or consensus or establish* or determin* or validat* or identif* or build* or implement* or defin* or introduc*) NEAR/4 ((“entrustable professional activit*” or (framework* NEAR/3 competenc*) or (standard* NEAR/3 competenc*) or (profile* NEAR/3 competenc*) or “core competenc*” or “competency model*” or “capability framework*”)))

OR

TI= ((develop* or produc* or map* or construct* or consensus or establish* or determin* or validat* or identif* or build* or implement* or defin* or introduc*) NEAR/4 ((“entrustable professional activit*” or (framework* NEAR/3 competenc*) or (standard* NEAR/3 competenc*) or (profile* NEAR/3 competenc*) or “core competenc*” or “competency model*” or “capability framework*”)))

AND

AB =(medicine or medical Or physician* Or pediatric* or paediatric* Or anaesthet* or dermatolog* Or “general practi*” Or intensivist* or “critical care” or oncolog* Or obstetric* Or gynaecolog* or gynecolog* Or opthamolog* Or neonatolog* Or cardiolog* Or endocrinolog* Or genetic* or gastroenterolog* Or haematolog* or hematolog* Or immunolog* Or allerg* Or nephrolog* Or neurolog* or rheumatolog* Or patholog* Or hepatolog* Or geriatric* Or psychiatr* Or radiolog* Or “nuclear medicine” or surg* Or neurosurg* Or urolog* Or Nurs* or midwif* Or midwives Or “art therap*” or audiolog* or radiograph* or “radiation therap*” or radiotherap* or “imaging technolog*” Or ultrasonograph* or sonograph* or dieti?ian* or dietetic* or “exercise physiolog*” or “music therap*” or “occupational therap*” or optometr* or orthopt* or orthoti* or prostheti* or osteopath* or perfusionist* or physiotherap* or “physical therap*” or podiatr* or psycholog* or “rehabilitation counsel*” or “social worker*” or “speech therap*” or “speech patholog*” or dent* or paramed* Or “emergency medical technician*” or pharmac*)

OR

TI =(medicine or medical Or physician* Or pediatric* or paediatric* Or anaesthet* or dermatolog* Or “general practi*” Or intensivist* or “critical care” or oncolog* Or obstetric* Or gynaecolog* or gynecolog* Or opthamolog* Or neonatolog* Or cardiolog* Or endocrinolog* Or genetic* or gastroenterolog* Or haematolog* or hematolog* Or immunolog* Or allerg* Or nephrolog* Or neurolog* or rheumatolog* Or patholog* Or hepatolog* Or geriatric* Or psychiatr* Or radiolog* Or “nuclear medicine” or surg* Or neurosurg* Or urolog* Or Nurs* or midwif* Or midwives Or “art therap*” or audiolog* or radiograph* or “radiation therap*” or radiotherap* or “imaging technolog*” Or ultrasonograph* or sonograph* or dieti?ian* or dietetic* or “exercise physiolog*” or “music therap*” or “occupational therap*” or optometr* or orthopt* or orthoti* or prostheti* or osteopath* or perfusionist* or physiotherap* or “physical therap*” or podiatr* or psycholog* or “rehabilitation counsel*” or “social worker*” or “speech therap*” or “speech patholog*” or dent* or paramed* Or “emergency medical technician*” or pharmac*)
